# Lifestyle, clinical, and occupational risk factors of recurrent stroke among the working-age group: A systematic review and meta-analysis

**DOI:** 10.1016/j.heliyon.2023.e13949

**Published:** 2023-02-22

**Authors:** Araya Chiangkhong, Charin Suwanwong, Yupha Wongrostrai

**Affiliations:** a Kuakarun Faculty of Nursing, Navamindradhiraj University, Bangkok, Thailand; b Behavioral Science Research Institute, Srinakharinwirot University, Bangkok, Thailand

**Keywords:** Recurrent stroke, Risk factors, Meta-analysis, Working-age group

## Abstract

**Background:**

Stroke recurrence is increasing in the working-age population. This study aimed to summarize and evaluate the risk factors for recurrent stroke among the working-age population.

**Methods:**

Relevant studies were extracted from several databases following Preferred Reporting Items for Systematic Reviews and Meta-Analyses guidelines. Fixed- or random-effects estimates of the pooled odds ratio (OR) and 95% confidence interval (CI) of risk factors for recurrent stroke were generated based on heterogeneity. Subgroup and publication bias analyses were also performed.

**Results:**

Seventeen studies were included in the meta-analysis. Pooled effects results revealed that the risk of recurrent stroke in the working-age group was as follows: Diabetes (OR = 1.85, 95% CI: 1.47, 2.32), hypertension (OR = 1.27, 95% CI: 1.12, 1.44), smoking (OR = 1.52, 95% CI: 1.27, 1.81), history of cardiac disease (OR = 2.86, 95% CI: 2.22, 3.67), history of stroke (OR = 2.45, 95% CI: 1.81, 3.31), and National Institutes of Health stroke severity score (OR = 1.09, 95% CI: 1.03, 1.15).

**Conclusion:**

These findings suggest that several factors, such as diabetes, hypertension, smoking, history of cardiac disease and stroke, and severity of a stroke, are potential risk factors for recurrent stroke in the working-age group. Therefore, strategies to reduce those risk factors should be adopted and attention paid to prevent recurrent stroke among working-age populations.

## 1 Introduction

Stroke is a major global public health concern for the 21st century, as the number of stroke patients is expected to rise in the future [1]. Stroke burden is associated with high mortality and morbidity; it is the second leading cause of death worldwide, with a mortality rate of 5.5 million per year [2].

Approximately half of the patients surviving an initial stroke have a significantly increased risk of recurrent stroke within 30 days to 5 years, which leads to increased mortality [[3], [4], [5]]. Recognizing warning signs, such as sudden unilateral weakness, difficulty speaking, difficulty walking, loss of balance, or dizziness [6], may reduce the time delay between the occurrence of an event and hospital admission, increasing the number of patients who receive better treatment and thereby preventing recurrent stroke [7]. Recurrent stroke has an increased risk of disability [8] and health care expenses cost more than the first stroke [9]. However, not all patients observe any warning signs.

Compared with the first stroke, recurrent stroke is more serious, difficult to treat, and causes mortality [10], especially in the working-age group, who have an increased incidence of stroke [11]. The working-age group has an increased risk of recurrent stroke owing to lifestyle factors, such as diabetes and hypertension [12], including occupational/work factors, such as blue- and white-collar groups [13], and clinical risk factors, such as previous history of stroke and myocardial infarction [14]. Generally, patients still may return to work, but those with more severe symptoms are unable to resume work [15]. Healthy lifestyles (daily fruit consumption, diet, good sleep, physical exercise, and smoking cessation) results in a higher quality of life, especially in working-age people after stroke [16], and a lower risk of recurrent stroke [17,18].

Recently, systematic reviews and meta-analyses investigated the association between risk factors and recurrent stroke [[19], [20], [21]]. However, no comprehensive systematic review has summarized the results among working-age individuals. Therefore, we conducted a systematic review and meta-analysis to summarize and evaluate lifestyle, clinical, and occupational risk factors for recurrent stroke in working-age people.

## 2 Methods

### 2.1 Search strategy and data sources

This review followed the Preferred Reporting Items for Systematic Reviews and Meta-Analyses (PRISMA) guidelines. PubMed, ScienceDirect, Google Scholar, SCOPUS, and the Thai Citation Index (TCI) were searched using medical subject headings (MeSH) or keywords. The following search terms were used: (recurrent OR recurrence OR relapse) AND (stroke) AND (factor OR predictor) AND (prospective study). The search was limited to English and Thai language articles.

### 2.2 Eligibility criteria and study selection

An initial eligibility screening of all titles and abstracts was performed, and studies reporting recurrent stroke were selected for further review. The following criteria were used for study selection: (1) studies reporting recurrent stroke after any subtype of stroke; (2) studies in adults aged 15–64 years, as this is considered the working-age population [22]; (3) studies using a prospective design; and (4) studies that provided an odds ratio (OR)/risk ratio (RR)/hazard ratio (HR) estimates. Recurrent stroke was defined as a focal neurological deficit lasting more than 24 h and occurring after the initial stroke. Patients with subsequent stroke within 24 h, experimental studies, systematic reviews, and meta-analyses were excluded. Two authors (CS and YW) independently examined eligible studies, and any disagreement was mediated by a third author (AC).

### 2.3 Data extraction and quality assessment

Data extraction was performed by two authors (CS and YW) and included first authors' name, year of publication, study location, age range, recurrence rate, stroke type, and factors of recurrent stroke. The Newcastle-Ottawa Scale (NOS) [23] was used to assess the methodological quality of all included studies, and any disagreement was resolved by discussion. Quality assessment scores were defined as poor (0–3), fair (4–6), or good (7–9).

### 2.4 Statistical analysis

The association of lifestyle and occupational factors with recurrent stroke was assessed using either OR/RR/HR with 95% confidence interval (CI). As the incidence of recurrent stroke was relatively low, OR/HR was close to RR and was assumed to be the same measure. In the meta-analysis, the fixed- or random-effects models were used to generate OR with 95% CI. Cochran’s Q test and *I*^2^ statistics were used to assess heterogeneity across studies [24]. We chose the random-effects model if the heterogeneity *I*^2^ statistic was significant; otherwise, the fixed-effects model was chosen. To identify potential sources of heterogeneity, subgroup analysis was performed based on the study location, recurrence rates, stroke type, and study quality. Begg’s rank correlation test was used to assess publication bias. All statistical analyses were performed using STATA 17 software (StataCorp, College Station, TX, USA).

## 3 Results

### 3.1 Included studies and study characteristics

**Fig. 1:**
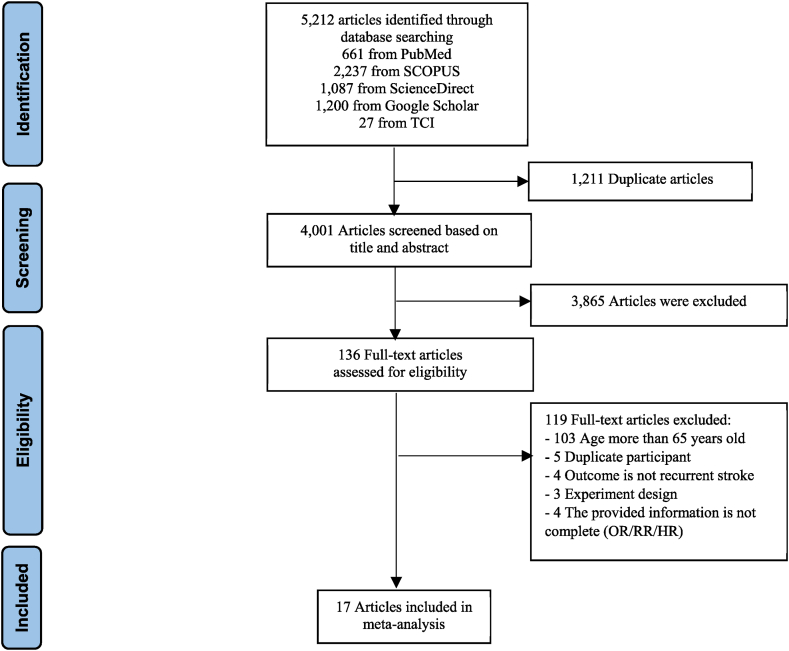
PRISMA flow diagram.

In this systematic review, we identified 5212 articles through database searches. We assessed the eligibility of 136 full-text articles. After evaluating full-text articles, 17 studies were included in the systematic review and meta-analysis (Fig. 1). The characteristics of the included studies are summarized in Table 1. These studies were published between 2008 and 2021. Of the 17 included studies, a total of 164,491 stroke patients were involved, and 3788 (2.3%) patients had recurrent stroke. Sixteen studies were cohort studies and one study used a case-control design. Ten studies were conducted in Western countries and 7 in Eastern countries. The studies were conducted across working ages ranging from 15 to 69 years. Stroke recurrence was reported to range from 0.3% to 36.2%. According to the NOS, 7 studies scored 7 or more (good), 9 studies scored 4–6 (fair), and only one study had poor quality.

**Table 1:** Summary of results.

First Author (year)	Study Location	Sample size	Cases of Recurrence	Recurrence Rate	Age Range	Stroke Type	Risk Factors of Recurrence Stroke	NOS
Jin (2018) [25]	USA	12,392	506	4.1%	18–45	Any	Diabetes	7
Arntz (2016) [26]	Netherlands	656	67	10.2%	18–50	IS	Diabetes, hypertension, smoking, history of cardiovascular disease	6
Musanoja (2016) [27]	Finland	1004	142	14.1%	15–49	IS	Hypertension	5
M’barek (2021) [28]	Tunisia	270	98	36.2%	18–50	IS	Hypertension, diabetes, heart failure, family history of stroke, NIHSS	5
Policardo (2015) [29]	Italy	43,332	1736	4.0%	16–69	IS	Diabetes	8
Putaala (2010) [30]	Finland	807	72	8.9%	15–49	IS	History of stroke, diabetes	9
Li (2017) [31]	China	1395	94	6.7%	18–45	IS	Atrial fibrillation, smoking, NIHSS	5
Han (2020) [32]	China	1185	273	23.0%	<65	Any	Diabetes	9
Zhao (2017) [33]	China	121	9	7.4%	18–45	IS	Diabetes	5
Yu (2021) [34]	USA	29,012	79	0.3%	18-44, >45	Any	Smoking, hypertension, diabetes, posttraumatic stress disorder	7
Li (2008) [35]	Sweden	69,625	275	0.4%	40–65	Any	Income	6
Elhefnawy (2021) [36]	Malaysia	1576	96	6.1%	18–59	IS	Ischemic heart disease, hypertension, hyperlipidemia	6
Yuan (2020) [37]	China	604	79	13.1%	18–49	IS	Hypertension, diabetes, smoking	5
Lank (2019) [38]	USA	663	96	14.6%	45–64	IS	Not having a primary care physician	8
Simonetti (2015) [39]	Switzerland	624	16	2.7%	16–55	Any	History of stroke	7
Fu (2015) [14]	China	1017	143	14.1%	18–64	IS	Hypertension, coronary heart disease, history of stroke	6
Divišová (2020) [40]	Czech Republic	208	7	3.4%	18–50	IS	Obesity	3

*Note:* IS = ischemic stroke, Any = any stroke type, NOS = Newcastle-Ottawa Scale, NIHSS: National Institutes of Health Stroke Severity.

### 3.2 Lifestyle risk factors

**Fig. 2:**
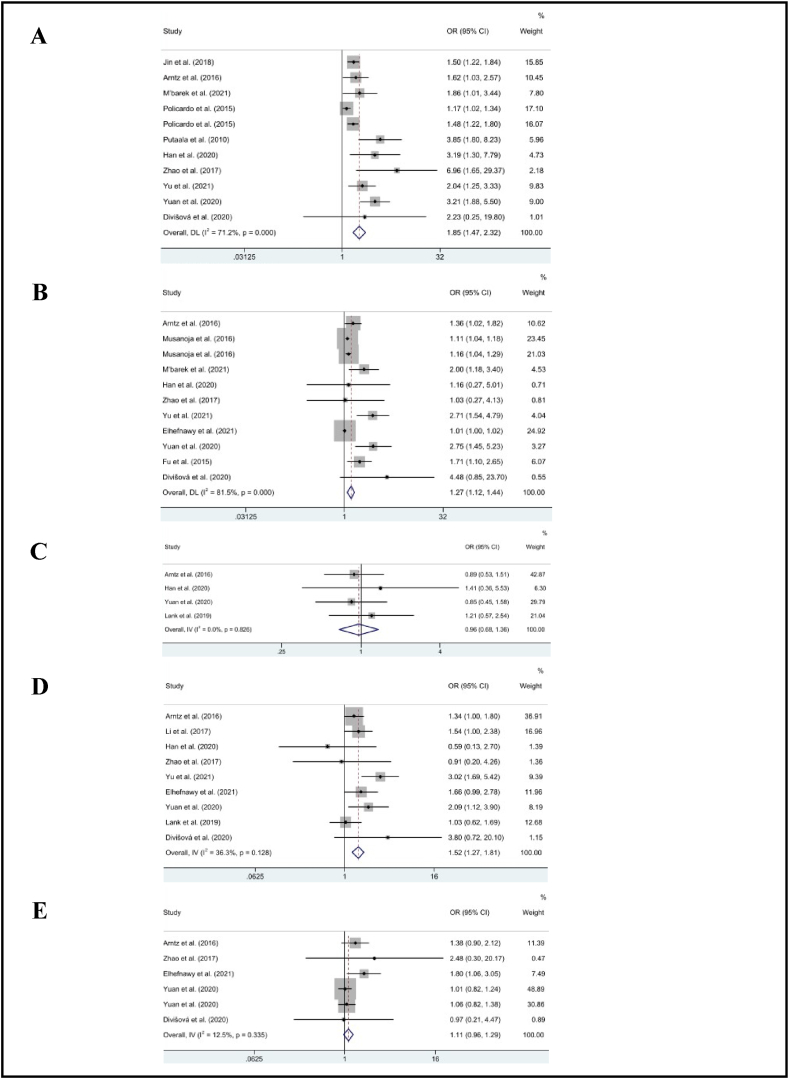
The pooled estimates of diabetes (A), hypertension (B), alcohol (C), smoking (D), and hyperlipidemia (E) on recurrent stroke.

As shown in Table 2, the pooled effect results illustrated a significant association between diabetes (OR = 1.85, 95% CI: 1.47, 2.32, *p* < .01; *I*^2^ = 71%), hypertension (OR = 1.27, 95% CI: 1.12, 1.44, *p* < .01; *I*^2^ = 82%), smoking (OR = 1.52, 95% CI: 1.27, 1.81, *p* < .01; *I*^2^ = 36%), and recurrent stroke in the working-age group (*p* < .01). However, alcohol consumption (OR = 0.96, 95% CI: 0.68, 1.36, *p* = .83; *I*^2^ = 0%) and hyperlipidemia (OR = 1.11, 95% CI: 0.96, 1.29, *p* = .15; *I*^2^ = 13%) were not associated with recurrent stroke [Fig. 2(A-E)].

**Table 2:** Pooled effect for risk factors of recurrent stroke among working-age group.

Lifestyle risk factors	No. of studies	OR (95% CI)	Z	*p-value*	*I*^2^
Diabetes	11	1.85 (1.47, 2.32)	5.32	<.01	71%
Hypertension	11	1.27 (1.12, 1.44)	3.74	<.01	82%
Smoking	9	1.52 (1.27, 1.81)	4.57	<.01	36%
Alcohol	4	0.96 (0.68, 1.36)	0.21	.83	0%
Hyperlipidemia	6	1.11 (0.96, 1.29)	1.46	.15	13%
**Medical history factors**	**No. of studies**	**OR (95% CI)**	**Z**	***p-value***	***I***^**2**^
Atrial fibrillation	3	1.86 (0.93, 3.69)	1.76	.08	0%
History of cardiac disease	8	2.86 (2.22, 3.67)	8.19	<.01	17%
History of stroke	3	2.45 (1.81, 3.31)	5.80	<.01	0%
NIHSS score	3	1.09 (1.03, 1.15)	3.00	<.01	47%
**Occupational risk factors**	**No. of studies**	**OR (95% CI)**	**Z**	***p-value***	***I***^**2**^
Self-employed	2	1.26 (0.63, 2.52)	0.66	.51	0%
Skilled manual	2	0.89 (0.49, 1.61)	0.39	.69	0%
Unskilled manual	2	1.08 (0.63, 1.85)	0.27	.79	30%
Low-level nonmanual	2	1.03 (0.58, 1.83)	0.11	.91	0%
Medium-level nonmanual	2	0.60 (0.31, 1.15)	1.54	.12	18%

### 3.3 Clinical risk factors

**Fig. 3:**
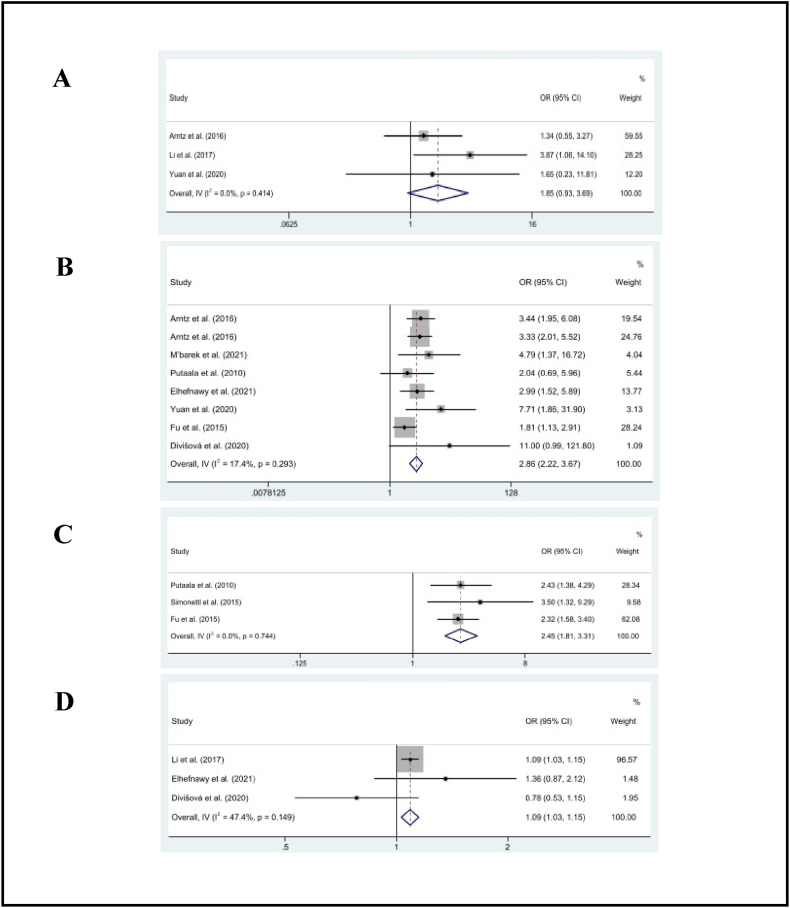
The pooled estimates of atrial fibrillation (A), history of cardiac disease (B), history of stroke (C), and NIHSS stroke severity (D) on recurrent stroke.

As shown in Table 2, the pooled effect results illustrated a significant association between history of cardiac disease (OR = 2.86, 95% CI: 2.22, 3.67, *p* < .01; *I*^2^ = 17%), history of stroke (OR = 2.45, 95% CI: 1.81, 3.31, *p* < .01; *I*^2^ = 0%), National Institutes of Health stroke severity (NIHSS) score (OR = 1.09, 95% CI: 1.03, 1.15, *p* < .01; *I*^2^ = 47%), and recurrent stroke in the working-age group (*p* < .01). However, atrial fibrillation (OR = 1.86, 95% CI: 0.93, 3.69, *p* = .08; *I*^2^ = 0%) was not associated with recurrent stroke [Fig. 3(A-D)].

### 3.4 Occupational risk factors

**Fig. 4:**
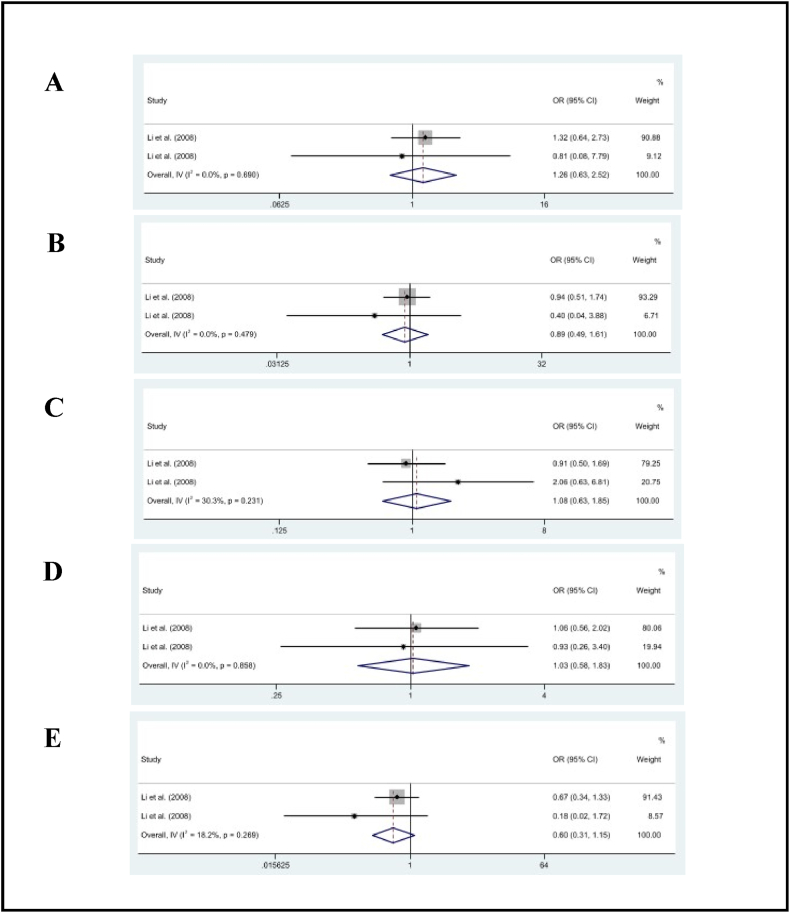
The pooled estimates of self-employed (A), skilled manual (B), nonskilled manual (C), low-level nonmanual (D), and medium-level nonmanual (E) on recurrent stroke.

As shown in Table 2, the pooled effect results showed no significant association between self-employed (OR = 1.26, 95% CI: 0.63, 2.52, *p* = .51; *I*^2^ = 0%), skilled manual (OR = 0.89, 95% CI: 0.49, 1.61, *p* = .69; *I*^2^ = 0%), unskilled manual (OR = 1.08, 95% CI: 0.63, 1.85, *p* = .79; *I*^2^ = 30%), low-level non-manual (OR = 1.03, 95% CI: 0.58, 1.83, *p* = .91; *I*^2^ = 0%), and medium-level non-manual (OR = 0.60, 95% CI: 0.31, 1.15, *p* = .12; *I*^2^ = 18%) workers and recurrent stroke [Fig. 4(A-E)].

### 3.5 Subgroup analysis

Diabetes and hypertension showed a high degree of heterogeneity (*I*^2^ > 50%). We then conducted a meta-regression analysis to check the sources of heterogeneity. Study location significantly explained the variance in the effects of diabetes on recurrent stroke (*p* < .05) and stroke type significantly explained the variance in the effects of hypertension on recurrent stroke (*p* < .01).

**Fig. 5:**
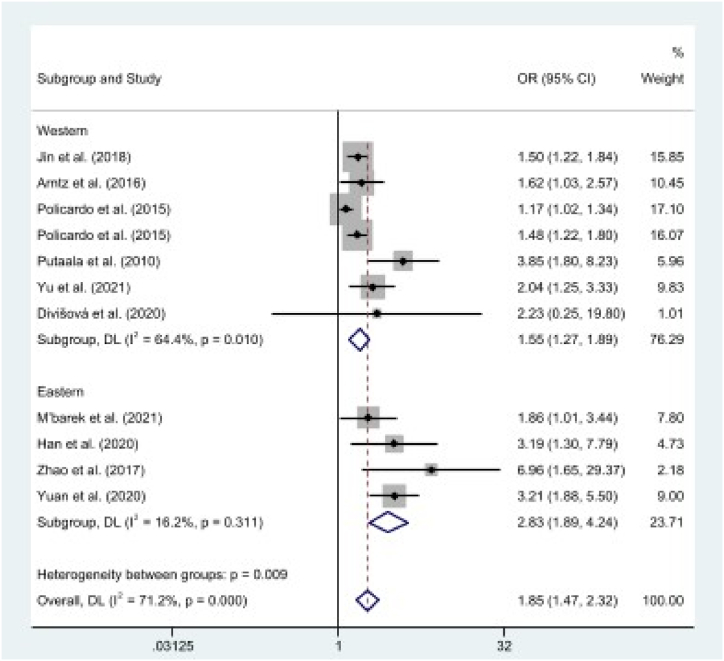
A subgroup analysis of diabetes on recurrent stroke based on study location, comparing Western populations and Eastern populations.

**Fig. 6:**
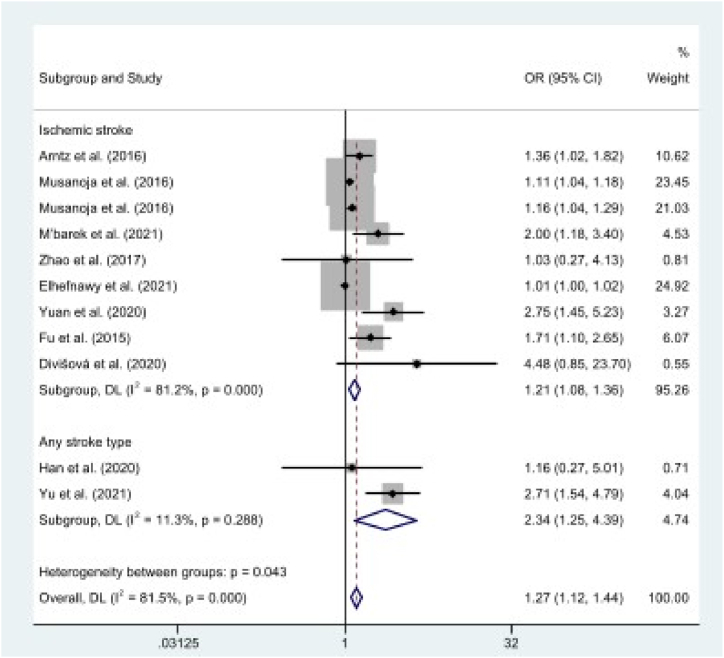
A subgroup analysis of diabetes on recurrent stroke based on stroke type, comparing ischemic stroke and any stroke type.

In subgroup analysis based on study location and stroke type (Table 3), the results indicated that diabetes was associated with the risk of recurrent stroke in both Eastern (OR = 2.83, 95% CI: 1.89, 4.24, *p* < .01; *I*^2^ = 16%) and Western populations (OR = 1.55, 95% CI: 1.27, 1.89, *p* < .01; *I*^2^ = 64%) (Fig. 5). In addition, the results indicated that hypertension was associated with the risk of recurrent stroke for both stroke types (OR = 2.34, 95% CI: 1.25, 4.39, *p* < .01; *I*^2^ = 11%) and ischemic stroke (OR = 1.21, 95% CI: 1.08, 1.36, *p* < .01; *I*^2^ = 81%) (Fig. 6).

**Table 3:** Subgroup analyses of risk factors and recurrent stroke.

Risk factors	Study Characteristics		No. of studies	OR (95% CI)	Z	*p-value*	*I*^*2*^
Diabetes	Study Location						
		Western	7	1.55 (1.27, 1.89)	4.25	<.01	64%
		Eastern	4	2.83 (1.89, 4.24)	5.04	<.01	16%
Hypertension	Stroke Type						
		IS	9	1.21 (1.08, 1.36)	3.20	<.01	81%
		Any	2	2.34 (1.25, 4.39)	2.65	<.01	11%

IS: ischemic stroke; Any: any stroke type, OR: odds ratio.

### 3.6 Publication bias

Based on the results of the Begg’s rank correlation test (diabetes, *p* = .06, hypertension, *p* = 1.00; smoking, *p* = .35, history of cardiac disease, *p* = .06, history of stroke, *p* = .30, NIHSS stroke severity, *p* = 1.00), there was no evidence of publication bias.

## 4 Discussion

This systematic review and meta-analysis evaluated various risk factors for recurrent stroke among working-age groups. Based on the 17 included studies, three lifestyle risk factors (diabetes, hypertension, and smoking) and three clinical risk factors (history of cardiac disease, history of stroke, and NIHSS stroke severity) were found to be associated with recurrent stroke in the working-age group. However, no association was found between occupational risk factors and recurrent stroke. According to the subgroup analysis, there was an association between recurrent stroke and diabetes or hypertension, regardless of study location or stroke type. Furthermore, there was no evidence of publication bias based on Begg’s rank correlation test.

Lifestyle risk factors such as diabetes, hypertension, and smoking were associated with recurrent stroke in the working-age group. These results were in line with previous studies that reported the risk of recurrent stroke associated with diabetes, hypertension, and smoking in young adults [26,37]. Healthy lifestyle was associated with reduced risk of recurrent stroke [18,41]. Moreover, pharmacological treatments (such as antihypertensive and antidiabetic medications) were associated with a lower risk of recurrent stroke [42,43]. Therefore, to reduce and prevent stroke recurrence in the working-age group, young adults need to be considered for treatment and lifestyle modification (such as antidiabetic medication, antihypertensive medication, smoking cessation, eating a healthy diet, and performing exercise activities) to reduce the risk of recurrent events. Clinical risk factors such as history of cardiac disease, history of stroke, and NIHSS stroke severity were associated with recurrent stroke in the working-age group. Consistent with previous studies, these clinical risk factors were associated with a higher risk of recurrent stroke in young adults [15]. Other researchers have also found that these clinical risk factors are associated with stroke recurrence among the general population, including older individuals, and are not specific to the working-age group. Fu et al. [15] also found that a previous history of stroke and coronary heart disease in the elderly was related to recurrent stroke. Wu et al. [44] showed an increased risk of recurrent stroke in patients with moderate and severe stroke relative to those with mild stroke. These results confirm that clinical risk factors (history of cardiac disease, previous stroke history, and stroke severity) could be used as predictors of recurrent stroke. There is no association between occupational status and recurrent stroke indicating there is no evidence to support an association between occupational risk factors and recurrent stroke in the working age group. A possible reason for this phenomenon is that young adults who have an occupation receive better healthcare benefits. Returning to work is the primary goal in the rehabilitation process of the working-age group [45]. However, a number of studies have investigated occupational risk factors and stroke risk. Long working hours [46] and stress at work [47] are associated with an increased risk of stroke. Perceived psychosocial work environment is also associated with an increased stroke risk [48]. However, it is reasonable to assume that occupational risk factors, especially long working hours, stress at work and psychosocial environment, may have affected the development of stroke recurrence in the working-age group.

The results of this study indicate that diabetes is a risk factor for recurrent stroke among working-age groups in both Western and Eastern populations. This finding is consistent with previous meta-analysis results; the effect of diabetes on stroke recurrence showed no regional differences [49]. From the results of our investigation, recurrent stroke associated with diabetes was greater among Eastern populations than among Western populations. A possible explanation for this phenomenon is that Eastern populations have small sample sizes; therefore, there is a possibility that the findings are affected by small-study effects [50]. Previous studies have shown that the prevalence of diabetes in stroke patients was relatively high in Eastern populations [[51], [52], [53]]. Therefore, further research is needed to explore the similarities and differences concerning findings observed in Western and Eastern populations.

Furthermore, this study found that hypertension is a risk factor for recurrent stroke among working-age groups for both stroke type and ischemic stroke. In fact, the risk of recurrent stroke associated with hypertension is greater among working-age groups with any type of stroke than among those with ischemic stroke. A possible reason for this phenomenon is that studies included any stroke type, including hemorrhagic stroke. Hemorrhagic strokes cause a severe increase in blood pressure [54] and lead to worse functional and clinical status compared to ischemic stroke [55,56]. A previous study found that the effect of hypertension on stroke was greater in hemorrhagic stroke than in ischemic stroke [57]. However, management of blood pressure in hemorrhagic stroke should be considered to prevent recurrent events [58,59]. Therefore, blood pressure-lowering treatment is an effective measure to reduce recurrent stroke in all stroke types.

However, this study had several limitations. First, most of the studies were conducted in high- and middle-income countries. Thus, more research evidence from low-income countries is required. Second, the recurrence rate in Eastern populations was significantly higher than that in Western populations (t = 2.252, *p* = .04). A possible reason is that the Eastern population used smaller cohorts than the Western population. Results bias may be relatively high for small cohorts, but lower for larger samples. Bias may also be due to loss to follow-up, which represents a threat to the internal validity of effect estimates from cohort studies [60]. Another possible reason is that larger samples increase the power of the study compared to smaller ones. The effect sizes of large samples have sufficient statistical power to become more significant than small ones [61]. Therefore, a larger cohort study should be conducted to obtain a more precise estimate. Third, the included studies reported early and late recurrence and type of recurrence, which might affect the results. It is suggested that the differences in early and late recurrence and type of recurrence should be explored in further studies to reduce heterogeneity. Finally, some studies were adjusted with control variables; hence, confounding factors in some studies might bias the true association.

## 5 Conclusion

This meta-analysis summarizes and evaluates lifestyle, clinical, and occupational risk factors for recurrent stroke among working-age individuals. Diabetes, hypertension, smoking, history of cardiac disease and stroke and the severity of a stroke are potential risk factors for recurrent stroke in working-age groups. Strategies for those risk factors should be adopted and focus on preventing recurrent stroke among the working-age population.

## Author contribution statement

All authors listed have significantly contributed to the development and the writing of this article.

## Funding statement

This work was supported by Navamindradhiraj University Research Fund (RESEARCH.NMU 62/2564).

## Data availability statement

Data will be made available on request.

## Declaration of interest's statement

The authors declare that they have no known competing financial interests or personal relationships that could have appeared to influence the work reported in this paper.

## Additional information

No additional information is available for this paper.

## References

[1] Donkor E.S. (2018). Stroke in the 21^st^ century: a snapshot of the burden, epidemiology, and quality of life. Stroke Res. Treat..

[2] WHO (2020). The Top 10 Causes of Death. https://www.who.int/news-room/fact-sheets/detail/the-top-10-causes-of-death.

[3] Arsava E.M., Kim G., Oliveira-Filho J., Gungor L., Noh H.J., Lordelo M.J. (2016). Prediction of early recurrence after acute ischemic stroke. JAMA Neurol..

[4] Khanevski A.N., Bjerkreim A.T., Novotny V., Naess H., Thomassen L., Logallo N. (2019). Recurrent ischemic stroke: incidence, predictors, and impact on mortality. Acta Neurol. Scand..

[5] Stahmeyer J.T., Stubenrauch S., Geyer S., Weissenborn K., Eberhard S. (2019). The frequency and timing of recurrent stroke: an analysis of routine health insurance data. Dtsch. Arztebl. Int..

[6] Saengsuwan J., Suangpho P., Tiamkao S. (2017). Knowledge of stroke risk factors and warning signs in patients with recurrent stroke or recurrent transient ischaemic attack in Thailand. Neurol. Res. Int..

[7] Soto-Cámara R., González-Bernal J.J., González-Santos J., Aguilar-Parra J.M., Trigueros R., López-Liria R. (2020). Knowledge on signs and risk factors in stroke patients. J. Clin. Med..

[8] Elwan M.E., Al-emam A.I., Munir A.N., Melake M.S. (2021). Does the second ischemic stroke herald a higher proportional risk for cognitive and physical impairment than the first-ever one? Egypt. J. Neurol. Psychiatry Neurosurg..

[9] Engel-Nitz N.M., Sander S.D., Harley C., Rey G.G., Shah H. (2010). Costs and outcomes of noncardioembolic ischemic stroke in a managed care population. Vasc. Health Risk Manag..

[10] Zhuo Y., Wu J., Qu Y., Yu H., Huang X., Zee B. (2020). Clinical risk factors associated with recurrence of ischemic stroke within two years: a cohort study. Medicine (Baltim.).

[11] Rosengren A., Giang K.W., Lappas G., Jern C., Torén K., Björck L. (2013). Twenty-four-year trends in the incidence of ischemic stroke in Sweden from 1987 to 2010. Stroke.

[12] Giang K.W., Björck L., Ståhl C.H., Nielsen S., Sandström T.Z., Jern C. (2016). Trends in risk of recurrence after the first ischemic stroke in adults younger than 55 years of age in Sweden. Int. J. Stroke.

[13] Zhu Y., Lu Y., Zhou M., Huang P., Zhang P., Guo Y. (2021). Occupational class differences in outcomes after ischemic stroke: a prospective observational study. BMC Publ. Health.

[14] Fu G., Yuan W., Du W., Yang Z., Fu N., Zheng H. (2015). Risk factors associated with recurrent stroke in young and elderly patients: a hospital-based study. Int. J. Gerontol..

[15] Westerlind E., Persson H.C., Sunnerhagen K.S. (2017). Return to work after a stroke in working age persons; a six-year follow up. PLoS One.

[16] Kowalczyk B., Zawadzka B. (2020). Lifestyle and quality of life in working-age people after stroke. Acta Clin. Croat..

[17] Hou L., Li M., Wang J., Li Y., Zheng Q., Zhang L. (2021). Association between physical exercise and stroke recurrence among first-ever ischemic stroke survivors. Sci. Rep..

[18] Huang Z., Yuan S., Li D., Hao H., Liu Z., Lin J. (2021). A nomogram to predict lifestyle factors for recurrence of large-vessel ischemic stroke. Risk Manag. Healthc. Pol..

[19] Kauw F., Takx R.A.P., de Jong H.W.A.M., Velthuis B.K., Kappelle L.J., Dankbaar J.W. (2018). Clinical and imaging predictors of recurrent ischemic stroke: a systematic review and meta-analysis. Cerebrovasc. Dis..

[20] Kolmos M., Christoffersen L., Kruuse C. (2021). Recurrent ischemic stroke – a systematic review and meta-analysis. J. Stroke Cerebrovasc. Dis..

[21] Zheng S., Yao B. (2019). Impact of risk factors for recurrence after the first ischemic stroke in adults: a systematic review and meta-analysis. J. Clin. Neurosci..

[22] OECD. Working (2022).

[23] Stang A. (2010). Critical evaluation of the Newcastle-Ottawa scale for the assessment of the quality of nonrandomized studies in meta-analyses. Eur. J. Epidemiol..

[24] Higgins J.P.T., Thompson S.G., Deeks J.J., Altman D.G. (2003). Measuring inconsistency in meta-analysis. BMJ.

[25] Jin P., Diaz I.M., Stein L., Thaler A., Tuhrim S., Dhamoon M.S. (2008). Intermediate risk of cardiac events and recurrent stroke after stroke admission in young adults. Int. J. Stroke.

[26] Arntz R.M., van Alebeek M.E., Synhaeve N.E., van Pamelen J., Maaijwee N.A., Schoonderwaldt H. (2016). The very long-term risk and predictors of recurrent ischemic events after a stroke at a young age: the FUTURE study. Eur. Stroke J..

[27] Mustanoja S., Putaala J., Gordin D., Tulkki L., Aarnio K., Pirinen J. (2016). Acute-phase blood pressure levels correlate with a high risk of recurrent strokes in young-onset ischemic stroke. Stroke.

[28] M'barek L., Sakka S., Megdiche F., Farhat N., Maalla K., Turki D. (2021). Traditional risk factors and combined genetic markers of recurrent ischemic stroke in adults. J. Thromb. Haemostasis.

[29] Policardo L., Seghieri G., Francesconi P., Anichini R., Franconi F., Seghieri C., Del Prato S. (2015). Gender difference in diabetes-associated risk of first-ever and recurrent ischemic stroke. J. Diabet. Complicat..

[30] Putaala J., Haapeniemi E., Metso A.J., Metso T.M., Artto V., Kaste M., Tatlisumak T. (2010). Recurrent ischemic events in young adults after first-ever ischemic stroke. Ann. Neurol..

[31] Li F., Yang L., Yang R., Xu W., Chen F., Li N., Zhang J. (2017). Ischemic stroke in young adults of Northern China: characteristics and risk factors for recurrence. Eur. Neurol..

[32] Han J., Mao W., Ni J., Wu Y., Liu J., Bai L. (2020). Rate and determinants of recurrence at 1 year and 5 years after stroke in a low-income population in rural China. Front. Neurol..

[33] Zhao M., Deng X., Gao F., Zhang D., Wang S., Zhang Y. (2017). Ischemic stroke in young adults with Moyamoya disease: prognostic factors for stroke recurrence and functional outcome after revascularization. World Neurosurg.

[34] Yu S., Alper H.E., Nguyen A., Maqsood J., Brackbill R.M. (2021). Stroke hospitalizations, posttraumatic stress disorder, and 9/11-related dust exposure: results from the World Trade Center Health Registry. Am. J. Ind. Med..

[35] Li C., Hedblad B., Rosvall M., Buchwald F., Khan F.A., Engström G. (2008). Stroke incidence, recurrence, and case-fatality in relation to socioeconomic position: a population-based study of middle-aged Swedish men and women. Stroke.

[36] Elhefnawy M.E., Ghadzi S.M.S., Tangiisuran B., Zainal H., Looi I., Ibrahim K.A. (2021). population-based study comparing predictors of ischemic stroke recurrence after index ischemic stroke in non-elderly adults with or without diabetes. Int. J. Gen. Med..

[37] Yuan K., Chen J., Xu P., Zhang X., Gong X., Wu M. (2020). A nomogram for predicting stroke recurrence among young adults. Stroke.

[38] Lank R.J., Lisabeth L.D., Sánchez B.N., Zahuranec D.B., Kerber K.A., Skolarus L.E. (2019). Recurrent stroke in midlife is associated with not having a primary care physician. Neurology.

[39] Simonetti B.G., Mono M., Huynh-Do U., Michel P., Odier C., Sztajzel R. (2015). Risk factors, aetiology and outcome of ischaemic stroke in young adults: the Swiss Young Stroke Study (SYSS). J. Neurol..

[40] Divišová P., Šaňák D., Král M., Bártková A., Hutyra M., Zapletalová J. (2020). Young cryptogenic ischemic stroke: a descriptive analysis of clinical and laboratory characteristics, outcomes and stroke recurrence. J. Stroke Cerebrovasc. Dis..

[41] Shani S.D., Varma R.P., Sarma P.S., Sylaja P.N., Kutty V.R. (2021). Life style and behavioural factors are associated with stroke recurrence among survivors of first episode of stroke: a case control study. J. Stroke Cerebrovasc. Dis..

[42] Katsanos A.H., Filippatou A., Manios E., Deftereos S., Parissis J., Frogoudaki A. (2017). Blood pressure reduction and secondary stroke prevention: a systematic review and metaregression analysis of randomized clinical trials. Hypertension.

[43] Lee M., Saver J.L., Liao H., Lin C., Ovbiagele B. (2017). Pioglitazone for secondary stroke prevention: a systematic review and meta-analysis. Stroke.

[44] Wu Q., Cui J., Xie Y., Wang M., Zhang H., Hu X., Jiang F. (2021). Outcomes of ischemic stroke and associated factors among elderly patients with large-artery atherosclerosis: a hospital-based follow-up study in China. Front. Neurol..

[45] Vestling M., Tufvesson B., Iwarsson S. (2003). Indicators for return to work after stroke and the importance of work for subjective well-being and life satisfaction. J. Rehabil. Med..

[46] Descatha A., Sembajwe G., Pega F., Ujita Y., Baer M., Boccuni F. (2020). The effect of exposure to long working hours on stroke: a systematic review and meta-analysis from the WHO/ILO Joint Estimates of the Work-related Burden of Disease and Injury. Environ. Int..

[47] Booth J., Connelly L., Lawrence M., Chalmers C., Joice S., Becker C., Dougall N. (2015). Evidence of perceived psychosocial stress as a risk factor for stroke in adults: a meta-analysis. BMC Neurol..

[48] Jood K., Karisson N., Medin J., Pessah-Rasmussen H., Wester P., Ekberg K. (2017). The psychosocial work environment is associated with risk of stroke at working age. Scand. J. Work. Environ. Health.

[49] Shou J., Zhou L., Zhu S., Zhang X. (2015). Diabetes is an independent risk factor for stroke recurrence in stroke patients: a meta-analysis. J. Stroke Cerebrovasc. Dis..

[50] Sterne J.A., Gavaghan D., Egger M. (2000). Publication and related bias in meta-analysis: power of statistical tests and prevalence in the literature. J. Clin. Epidemiol..

[51] Lau L., Lew J., Borschmann K., Thijs V., Ekinci E.I. (2019). Prevalence of diabetes and its effects on stroke outcomes: a meta-analysis and literature review. J. Diabetes Investig..

[52] Lin X., Xu Y., Pan X., Xu J., Ding Y., Sun X. (2020). Global, regional, and national burden and trends of diabetes in 195 countries and territories: an analysis from 1990 to 2025. Sci. Rep..

[53] Szlachetka W.A., Pana T.A., Tiamkao S., Clark A.B., Kongbunkiat K., Sawanyawisuth K. (2020). Impact of diabetes on complications, long term mortality and recurrence in 608,890 hospitalised patients with stroke. Glob Heart.

[54] Lattanzi S., Cagnetti C., Provinciali L., Silvestrini M. (2017). How should we lower blood pressure after cerebral hemorrhage? a systematic review and meta-analysis. Cerebrovasc. Dis..

[55] Ojaghihaghighi S., Vahdati S.S., Mikaeilpour A., Ramouz A. (2017). Comparison of neurological clinical manifestation in patients with hemorrhagic and ischemic stroke. World J. Emerg. Med..

[56] Salvadori E., Papi G., Insalata G., Rinnoci V., Donnini I., Martini M. (2021). Comparison between ischemic and hemorrhagic strokes in functional outcome at discharge from an intensive rehabilitation hospital. Diagnostics (Basel).

[57] Tsai C., Anderson N., Thomas B., Sudlow C.L.M. (2016). Comparing risk factor profiles between intracerebral hemorrhage and ischemic stroke in Chinese and White populations: systematic review and meta-analysis. PLoS One.

[58] Li L., Zuurbier S.M., Kuker W., Warlow C.P., Rothwell P.M. (2021). Blood pressure control and recurrent stroke after intracerebral hemorrhage in 2002 to 2018 versus 1981 to 1986: population-based study. Stroke.

[59] Wajngarten M., Silva G.S. (2019). Hypertension and stroke: update on treatment. Eur. Cardiol..

[60] Howe C.J., Cole S.R., Lau B., Napravnik S., Eron J.J. (2016). Selection bias due to loss to follow up in cohort studies. Epidemiology.

[61] Schäfer T., Scwarz M.A. (2019). The meaningfulness of effect sizes in psychological research: differences between sub-disciplines and the impact of potential biases. Front. Psychol..

